# Outcomes Following Distal Nerve Blocks for Open Carpal Tunnel Release: A Single-Institution Retrospective Study

**DOI:** 10.7759/cureus.41258

**Published:** 2023-07-01

**Authors:** Paige S Tsuda, Austin L Du, Rodney A Gabriel, Brian P Curran

**Affiliations:** 1 Anesthesiology, Division of Regional Anesthesia, University of California San Diego, La Jolla, USA

**Keywords:** retrospective observational study, postoperative complications, carpal tunnel syndrome, ultrasound-guided regional anesthesia, carpal tunnel release

## Abstract

Background

Severe cases of carpal tunnel syndrome (CTS) are treated with surgical decompression, for which regional nerve blocks are often administered. There is little data about complications associated with these regional techniques for this surgery. The primary objective was to assess the association of ultrasound-guided regional anesthesia nerve blocks in patients undergoing carpal tunnel release with symptom resolution.

Methods

This single-institution, retrospective study analyzed all patients undergoing open carpal tunnel release from March 2018 to November 2020. Primary exposure was either regional anesthesia (median and ulnar nerve blocks) or non-regional anesthesia (general anesthesia or local infiltration by surgeon). The primary outcome measurement was symptom resolution at postoperative follow-up at 30-60 days. Secondary outcomes were postoperative surgical site infection, time in operating room (minutes), and post-anesthesia care unit (PACU) length of stay (min). The primary outcome was analyzed using multivariable logistic regression.

Results

A total of 417 patients were included in this study. Of these, 269 (64.5%) subjects received regional anesthesia as their primary anesthetic. When adjusting for confounders, the use of regional anesthesia was not associated with symptoms not improving at postoperative visit (OR 0.52, 95% CI 0.22 - 1.26, P = 0.15), postoperative surgical site infection (OR 1.47, 95% CI 0.44 - 4.85, p = 0.53), or operating room time duration (p = 0.09). However, the use of regional anesthesia was associated with an approximately 15-minute decrease in PACU length of stay (p < 0.001).

Conclusions

Regional anesthesia is a safe, effective, and time-efficient method for anesthesia in patients undergoing open carpal tunnel release.

## Introduction

Carpal tunnel syndrome (CTS) is a common neuropathic disorder caused by compression of the median nerve as it passes through the carpal tunnel [1]. It can cause debilitating pain and numbness in the median nerve distribution of the hand. CTS affects approximately 1-7% of the population, affecting more women than men [1,2]. Risk factors include, but are not limited to, obesity, diabetes, pregnancy, hypothyroidism, and prior wrist trauma [3].

Mild CTS can be treated conservatively with physical therapy, wrist splints, and corticosteroid injections [1]. More severe cases of CTS require surgical decompression, either endoscopically or with open surgery. Adequate anesthesia for carpal tunnel release surgery can be achieved in a variety of ways: local anesthetic infiltration, regional anesthesia, and general anesthesia. A regional anesthetic technique can be performed using either an intravenous block, also known as a Bier block, or with nerve blocks by anesthetizing the brachial plexus proximally or more distally at the level of the branches [4]. Distal nerve blocks for carpal tunnel release involve surrounding local anesthetic around the median and ulnar nerves, typically using ultrasound guidance.

There is inherent risk of nerve damage with all regional anesthesia techniques because of the close proximity of needle and local anesthetic to nerves [5]. The potential risk of neuropathy following regional anesthesia in patients with pre-existing nerve injury has been debated for decades [6]. This controversy applies particularly to carpal tunnel release surgery given the prevalence of pre-existing nerve damage in CTS. Commonly cited literature has warned of temporary post-procedural neuropraxias in healthy individuals [7], and cases of median nerve injury have been reported [8]. However, more modern studies have yet to identify definitive associations between regional anesthesia and neuropathy following carpal tunnel release [9-11]. The primary objective of this study is to provide some insight into if regional anesthesia nerve blocks in patients undergoing carpal tunnel release are safe, despite pre-existing nerve injury.

## Materials and methods

Study sample

This retrospective study was approved by our institution’s Human Research Protections Program for the collection of data from our electronic medical record system and the informed consent requirement was waived. Data were manually collected retrospectively from the electronic medical record system by one clinician from our healthcare system. Data from all patients that underwent open carpal tunnel release from March 2018 to November 2020 were extracted. Cases that included a concomitant surgery were excluded. The manuscript adheres to the applicable EQUATOR guidelines for observational studies.

Primary objective and data collection

The primary outcome measurement was symptom resolution at orthopedic surgery clinic postoperative follow-up at 30-60 days. Clinic notes were extracted and reviewed for symptoms before and after surgery. Symptoms included numbness, pain, and tingling. Each patient's outcome was assigned according to symptoms at follow-up relative to their presenting symptoms. Outcomes were categorized as complete resolution, some improvement, no improvement, or worsening symptoms. The outcome was converted to a binary variable in which patients either had symptoms improved (complete resolution or some improvement) or no symptom resolution (no improvement or worsening symptoms). Secondary outcomes of interest were postoperative surgical site infection, time in the operating room (minutes), and post-anesthesia care unit (PACU) length of stay (min). Time in the operating room was defined as the time between patient entering the operating room and leaving the operating room. Among patients under general anesthesia, operating room time includes anesthesia induction time since patients at our institution are induced in the operating room. At our institution, PACU discharge is standardized as the time at which patients affirm that their pain and nausea is adequately controlled. The primary independent variable was the use of ultrasound-guided regional anesthesia as the primary anesthetic. Patients either received: 1) regional anesthesia blocking the median and ulnar nerves at the level of the mid-forearm with bupivacaine 0.5% with 1:400,000 epinephrine (10mL per nerve) or lidocaine 2% with 1:400,000 epinephrine (10mL per nerve) or 2) non-regional anesthesia using general anesthesia with supraglottic airway or surgeon-applied superficial local infiltration with upper extremity tourniquet and monitored anesthesia care. Furthermore, we collected data on patient sex, age (years), body mass index (BMI), and American Society of Anesthesiologists physical status classification score (ASA PS). ASA PS was a binary variable in which patients either were ASA PS < 3 or ASA PS ≥ 3.

Statistical analysis

All statistical analysis was performed using R (version 3.6.1; R Foundation for Statistical Computing, Vienna, Austria). To compare baseline patient characteristics, we used Welch two-sample t-test and Pearson’s chi-square test for continuous and categorical variables, respectively. A p-value of <0.05 was considered statistically significant. The primary outcome of interest was symptom resolution. For binary outcomes (symptom resolution and postoperative surgical site infection), we used multivariable logistic regression to assess the association of the use of regional anesthesia vs non-regional anesthesia with the outcomes. In this model, we controlled for sex, age, BMI, and ASA PS. Here, we reported odds ratios (OR) and 95% confidence interval (CI), in which P <0.05 was considered statistically significant. For continuous outcomes (operating room minutes and PACU length of stay minutes), multivariable linear regression was used to assess the association of regional anesthesia with these operating room time metrics. In this model, we controlled for sex, age, BMI, and ASA PS. We report the coefficient and standard error for regional anesthesia from the model. For a power of 80%, the study would require 868 patients (434 per group) to detect a difference between 10% versus 5%. A P <0.05 was considered statistically significant.

## Results

During the study period, there were 428 subjects who underwent open carpal tunnel release. Eleven of these patients underwent a concomitant surgery and was therefore removed from the analysis, leaving a final sample size of 417 subjects. Of these, 269 (64.5%) subjects received regional anesthesia as their primary anesthetic. There was no difference between the regional and non-regional anesthesia cohorts based on ASA PS class, sex, age, or BMI (Table 1).

**Table 1:** Comparison of baseline patient characteristics in the non-regional versus regional anesthesia cohorts. ASA PS = American Society of Anesthesiologists Physical Status score, SD = standard deviation

	Non-regional Anesthesia		Regional Anesthesia		
Characteristics	n	%	n	%	p-value
Total	148	-	269	-	
ASA PS ≥ 3	49	33.1	93	34.6	0.85
Male Sex	53	35.8	108	40.1	0.44
Mean Age (years) [SD]	59.6 [14.9]		59.4 [14.5]		0.86
Mean BMI (kg/m^2^) [SD]	30.0 [7.3]		30.9 [6.8]		0.22

Twenty-two (5.3%) patients either reported no improvement or worsening symptoms of their carpal tunnel syndrome at postoperative orthopedic clinic follow-up. On univariate analysis, among patients that did not receive regional anesthesia or did receive regional anesthesia, 11 (7.4%) and 11 (4.1%) subjects, respectively, reported no improvement or worsening symptoms (p = 0.22). Furthermore, there was no difference in postoperative surgical site infection between both cohorts (p = 0.07). In regards to operating room efficiency metrics, there was no difference in mean operating room time between both cohorts (39.9 vs 37.3 minutes in the non-regional versus regional anesthesia cohorts, respectively, p = 0.12). The mean PACU length of stay in the non-regional versus regional anesthesia cohorts was 76.1 vs 61.7 minutes, respectively (p < 0.001) (Table 2).

**Table 2:** Comparison of outcomes between the non-regional versus regional anesthesia cohorts. CTS = carpal tunnel surgery; PACU = post-anesthesia care unit; SD = standard deviation; SSI = surgical site infection

	Non-regional anesthesia		Regional anesthesia		
Outcome	n	%	n	%	p-value
CTS symptoms did not improve at follow-up	11	7.4	11	4.1	0.22
Postoperative SSI	4	2.7	10	3.7	0.07
Mean Operating Room Time Duration (min) [SD]	39.9 [13.9]		37.3 [20.1]		0.12
Mean PACU Length of Stay (min) [SD]	76.1 [32.5]		61.7 [22.2]		<0.001

When adjusting for age, sex, BMI, and ASA PS using regression analysis, the use of regional anesthesia was not associated with symptoms not improving at postoperative visit (OR 0.52, 95% 0.22 - 1.26, p = 0.15), postoperative surgical site infection (OR 1.47, 95% CI 0.44 - 4.85, p = 0.53), or operating room time duration (coefficient = -3.04min, standard error = 1.84 min, p = 0.09). However, the use of regional anesthesia was associated with an approximately 15-minute decrease in PACU length of stay (coefficient = -14.5min, standard error = 2.7min, p < 0.001) (Table 3).

**Table 3:** Effects of regional anesthesia vs non-regional anesthesia on patient outcomes and theatre utilization Statistics presented are odds ratios and coefficients of each outcome among patients receiving regional anesthesia when compared to patients receiving non-regional anesthesia. CI = confidence interval; CTS = carpal tunnel surgery; PACU = post-anesthesia care unit; OR = odds ratio; SD = standard deviation; SSI = surgical site infection

Patient Outcome	OR (95% CI)	p-value
CTS symptoms did not improve at follow-up	0.52 [0.22 - 1.26]	0.15
Postoperative SSI	1.47 [0.44 - 4.85]	0.53
	Coefficient (Standard Error)	p-value
Operating Room Time (min)	-3.04 [1.84]	0.09
PACU Length of Stay (min)	-14.46 [2.70]	<0.001

## Discussion

The majority of patients undergoing carpal tunnel release surgery at our institution were given regional anesthesia as their primary anesthetic. At the postoperative orthopedic checkup, there was no difference in worsening or unchanged symptoms among patients who received regional anesthesia compared to those who did not. There was also no difference in postoperative surgical site infections or mean operating room time. Notably, PACU length of stay was significantly shorter for patients who received regional anesthesia compared to those who did not.

These results are consistent with other studies comparing regional anesthesia to non-regional techniques for carpal tunnel release. Parallel to our findings on post-operative symptoms, Nabhan and colleagues found no differences in long-term functional outcomes between patients who received local anesthesia and those who received intravenous regional anesthesia for carpal tunnel release surgery [9]. When comparing regional anesthesia and general anesthesia, Droog and colleagues similarly concluded that regional anesthesia was not associated with a higher risk of new-onset nerve injury following carpal tunnel release [11]. In both studies, carpal tunnel release with regional anesthesia showed no increased risk for new-onset procedure-induced deficits in the hand or wrist.

A notable benefit of regional anesthesia with carpal tunnel release surgery is the reduced PACU length of stay. Our study showed that patients who received regional anesthesia were discharged from the PACU approximately 15 minutes earlier than those who received other forms of anesthesia. Decreased PACU length of stay can reduce cost, increase patient satisfaction, and increase PACU efficiency [12]. This is particularly important in the setting of outpatient surgical centers due to the high volume and turnover of less time-consuming cases, such as carpal tunnel releases. New methods in regional anesthesia for outpatient hand surgeries have continued to decrease time to discharge without neglecting pain control [13].

Surgical literature has long debated whether regional anesthesia in populations with pre-existing neuropathy may lead to increased risk of post-operative nerve damage [14]. Early studies conducted prior to 1985 found rates of post-block neuropathy between 0.36% and 2.7% when paresthesias were provoked prior to anesthetic infusion. Many studies since then have been limited by difficulties in qualitatively separating anesthesia-induced neuropathy, surgery-induced neuropathy, and exacerbation of pre-existing neuropathic disorders. The more recent development of local anesthetic techniques has contributed towards the increased use of local analgesic infiltration rather than regional anesthesia [15]. However, the results of this retrospective study showed that regional anesthesia is not associated with worse patient outcomes in carpal tunnel release surgery. Patients with pre-existing neuropathy, as in CTS, did not report worsened symptoms post-operatively compared to patients who did not receive regional anesthesia. These findings suggest that regional anesthesia may be used safely in patients with CTS who require carpal tunnel release surgery.

There are several limitations to this study, mainly due to the retrospective nature of the analysis. There may be several confounders not accounted for in the study, which would be remedied by a randomized prospective clinical trial. Furthermore, we relied on clinician notes during the postoperative visit to ascertain symptom improvement of the CTS. Similarly, interpretation of symptom improvement is complicated by patients' and clinicians' limited ability to differentiate between surgery- and anesthesia-induced pain versus inadequate carpal decompression. Future studies should perform sensory tests to better qualify and quantify postoperative recovery. Importantly, the study may have been underpowered to detect a difference in our primary outcome. With our current sample size, a post-hoc power analysis demonstrated a power of 31.6% to detect a difference if we assume an alpha of 0.05 and incidence of non-symptom resolution of 7.4% and 4.1% (based on the reported rates in our study) in the non-regional versus regional anesthesia cohort, respectively. With a power of 80%, 1,560 patients (780 per group) would be required to identify the difference in symptom resolution found in our study. Nonetheless, we demonstrated that among 269 patients that did receive a peripheral nerve block (over three years), there were no differences in symptom resolution. This suggests that, given this current surgical volume, the use of nerve blocks is likely safe.

## Conclusions

In conclusion, regional anesthesia can provide adequate anesthesia with improved PACU length of stay in patients undergoing carpal tunnel surgery. Our retrospective analysis shows no increased risk of worse outcomes in patients who receive regional anesthesia compared to those who received local infiltration or general anesthesia.

## References

[1] Ashworth NL (2016). Carpal tunnel syndrome. Am Fam Physician.

[2] Dale AM, Harris-Adamson C, Rempel D (2013). Prevalence and incidence of carpal tunnel syndrome in US working populations: pooled analysis of six prospective studies. Scand J Work Environ Health.

[3] Geoghegan JM, Clark DI, Bainbridge LC, Smith C, Hubbard R (2004). Risk factors in carpal tunnel syndrome. J Hand Surg Br.

[4] Brown EM, McGriff JT, Malinowski RW (1989). Intravenous regional anaesthesia (Bier block): review of 20 years' experience. Can J Anaesth.

[5] Hogan QH (2008). Pathophysiology of peripheral nerve injury during regional anesthesia. Reg Anesth Pain Med.

[6] Lirk P, Birmingham B, Hogan Q (2011). Regional anesthesia in patients with preexisting neuropathy. Int Anesthesiol Clin.

[7] Urban MK, Urquhart B (1994). Evaluation of brachial plexus anesthesia for upper extremity surgery. Reg Anesth.

[8] Kim TH, Kim CK, Lee KD, Koo JH, Song SH (2014). Median nerve injury caused by brachial plexus block for carpal tunnel release surgery. Ann Rehabil Med.

[9] Nabhan A, Steudel WI, Dedeman L, Al-Khayat J, Ishak B (2011). Subcutaneous local anesthesia versus intravenous regional anesthesia for endoscopic carpal tunnel release: a randomized controlled trial. J Neurosurg.

[10] da Costa VV, de Oliveira SB, Fernandes Mdo C, Saraiva R (2011). Incidence of regional pain syndrome after carpal tunnel release. Is there a correlation with the anesthetic technique?. Rev Bras Anestesiol.

[11] Droog W, Lin DY, van Wijk JJ, Ho-Asjoe RC, Coert JH, Stolker RJ, Galvin EM (2019). Is it the surgery or the block? Incidence, risk factors, and outcome of nerve injury following upper extremity surgery. Plast Reconstr Surg Glob Open.

[12] Sarin P, Philip BK, Mitani A, Eappen S, Urman RD (2012). Specialized ambulatory anesthesia teams contribute to decreased ambulatory surgery recovery room length of stay. Ochsner J.

[13] Vaughn N, Rajan N, Darowish M (2020). Intravenous regional anesthesia using a forearm tourniquet: a safe and effective technique for outpatient hand procedures. Hand (N Y).

[14] Sorenson EJ (2008). Neurological injuries associated with regional anesthesia. Reg Anesth Pain Med.

[15] Al Youha S, Lalonde DH (2014). Update/Review: changing of use of local anesthesia in the hand. Plast Reconstr Surg Glob Open.

